# Epigenetic Silencing of Nucleolar rRNA Genes in Alzheimer's Disease

**DOI:** 10.1371/journal.pone.0022585

**Published:** 2011-07-22

**Authors:** Maciej Pietrzak, Grzegorz Rempala, Peter T. Nelson, Jing-Juan Zheng, Michal Hetman

**Affiliations:** 1 Department of Neurological Surgery and Kentucky Spinal Cord Injury Research Center, University of Louisville, Louisville, Kentucky United States of America; 2 Department of Pharmacology and Toxicology, University of Louisville, Louisville, Kentucky United States of America; 3 Department of Biostatistics, Medical College of Georgia, Augusta, Georgia, United States of America; 4 Sanders-Brown Center on Aging, University of Kentucky, Lexington, Kentucky, United States of America; Deutsches Krebsforschungszentrum, Germany

## Abstract

**Background:**

Ribosomal deficits are documented in mild cognitive impairment (MCI), which often represents an early stage Alzheimer's disease (AD), as well as in advanced AD. The nucleolar rRNA genes (rDNA), transcription of which is critical for ribosomal biogenesis, are regulated by epigenetic silencing including promoter CpG methylation.

**Methodology/Principal Findings:**

To assess whether CpG methylation of the rDNA promoter was dysregulated across the AD spectrum, we analyzed brain samples from 10 MCI-, 23 AD-, and, 24 age-matched control individuals using bisulfite mapping. The rDNA promoter became hypermethylated in cerebro-cortical samples from MCI and AD groups. In parietal cortex, the rDNA promoter was hypermethylated more in MCI than in advanced AD. The cytosine methylation of total genomic DNA was similar in AD, MCI, and control samples. Consistent with a notion that hypermethylation-mediated silencing of the nucleolar chromatin stabilizes rDNA loci, preventing their senescence-associated loss, genomic rDNA content was elevated in cerebrocortical samples from MCI and AD groups.

**Conclusions/Significance:**

In conclusion, rDNA hypermethylation could be a new epigenetic marker of AD. Moreover, silencing of nucleolar chromatin may occur during early stages of AD pathology and play a role in AD-related ribosomal deficits and, ultimately, dementia.

## Introduction

Regulation of gene expression is achieved through genetic and epigenetic mechanisms. While the former are strictly determined by the DNA sequence, the latter are not [1]. Instead, they involve chemical modifications of DNA and/or histones. Cytosine methylation at DNA CpG sites is the principal epigenetic modification of DNA that silences gene expression [2]. Although epigenetic regulation may be a part of genetically-determined programs such as X chromosome inactivation, it may also mediate the effects of environmental factors [1], [2]. For instance, in newborn rats, maternal care determines their adult stress sensitivity by reducing methylation of the glucocorticoid receptor gene promoter in the brain [3]. Hence, epigenetic dysregulation is an attractive candidate mechanism, by which extrinsic factors interact with genes to modulate normal or pathologic gene expression.

Epigenetic modulation has been proposed in the sporadic late-onset Alzheimer's disease (AD) [4]. However, of 62 loci whose methylation pattern was compared in AD- and control cerebral cortical samples, there were only two, *S100A2* and *SORBS3*, which showed changes in AD [5], [6]. While links between these two genes and AD pathology are unclear, none of the loci that are directly involved in AD pathogenesis appeared differentially methylated [5], [6]. Instead, AD was associated with increased methylation variability at 12 such loci [6]. However, the contribution of such an epigenetic “drift” to AD pathogenesis has not been yet tested.

The expression of repeated nucleolar rRNA genes (rDNA) initiates ribosomal biogenesis and is a subject of complex regulation including transcriptional silencing via dynamic methylation of 26 CpG sites in the rDNA promoter [7], [8]. The nucleolar repressive complex (NoRC) increases methylation of these sites inducing formation of silent chromatin, reducing rDNA transcription and down-regulating ribosomal biogenesis [7], [9]. Conversely, NoRC-mediated silencing promotes stability of rDNA, presumably by suppressing recombination [10].

The ribosome is the nexus for all cellular protein translation and reduced ribosomal activity has been suggested to be a major contributor to the pathology of Alzheimer's disease (AD) [11], [12], [13], [14]. Ribosomal depletion and accumulation of oxidized rRNA have been observed in the AD cerebral cortex [11], [12], [13]. In addition, stereological studies of hippocampal neurons revealed AD-associated reduction in nucleolar volume [15]. Conversely, in subjects with moderate AD pathology without cognitive impairment, nucleolar hypertrophy has been observed in both cortical and hippocampal neurons [15], [16]. Finally, in rat and mouse neurons, blocking nucleolar transcription induces neurodegeneration including neuronal death [17], [18], [19]. These results suggest that perturbed ribosomal biogenesis contributes to the AD pathogenesis. Therefore, the current study has been initiated to determine whether dysregulation of rDNA methylation occurs in AD.

## Results

### AD-associated increase in methyl-cytosine content in the rDNA promoter

**Figure 1:**
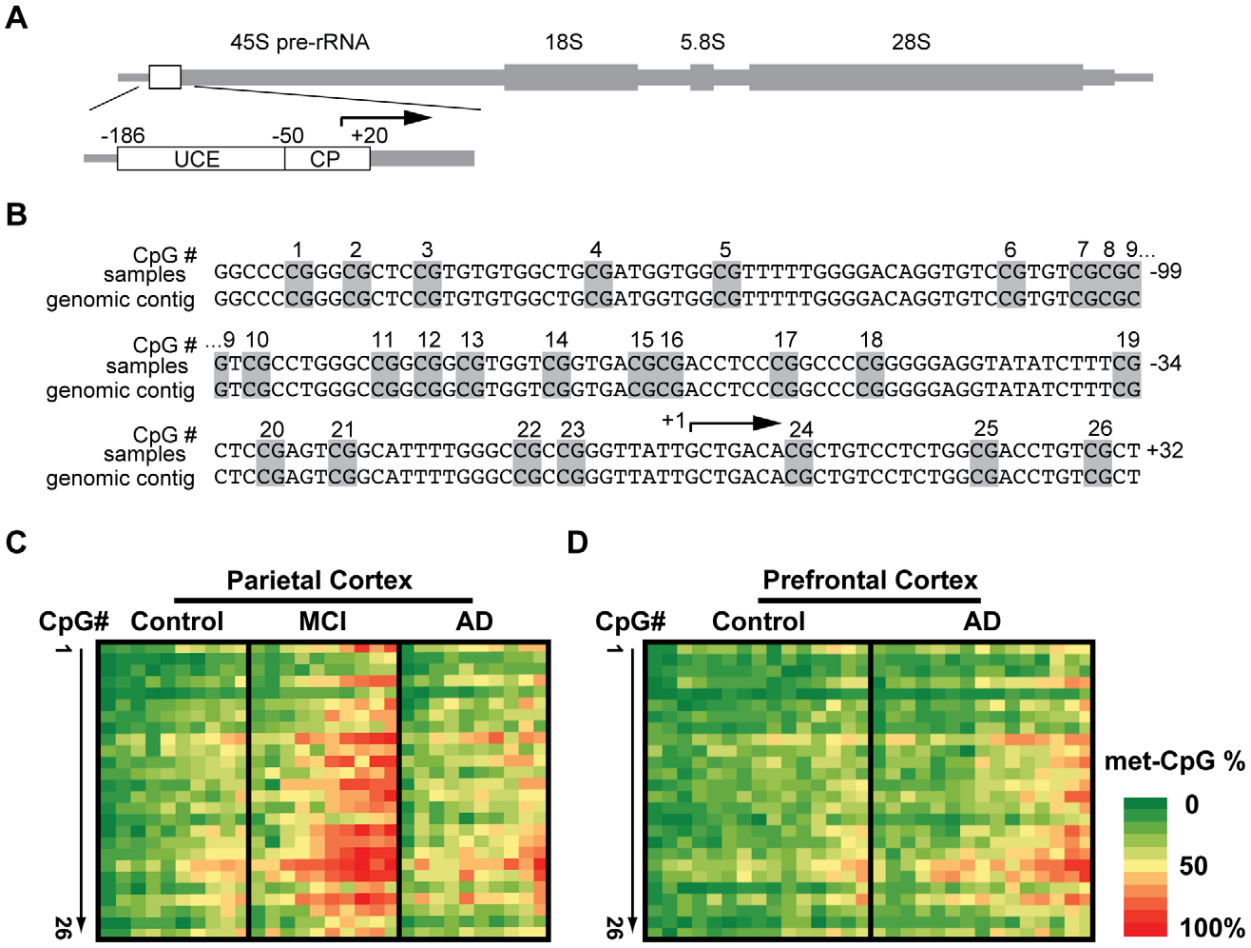
Methyl-cytosine (mC) mapping of the rDNA promoter region. ***A***, The rDNA gene unit. The location of the promoter region that was selected for bisulfite mapping of methyl cytosines is indicated by a box. That analyzed region included the Upstream Control Element (UCE) and the Core Promoter (CP) which contain 26 CpG methylation sites that are critical for epigenetic regulation of nucleolar transcription (for more detail, see Fig. S1). ***B***. Nucleotide sequence of the human rDNA promoter. The numbers on the right indicate positions relative to the transcription start site at position +1 (arrow); Potentially methylated CpG dinucleotides are marked grey and numbered. ***C***, Bisulfite sequencing analysis of parietal cortex from control-, MCI-, and AD individuals (n = 10 for each group). ***D***. Bisulfite sequencing analysis of prefrontal cortex from control-, AD individuals (n = 15 for each group). In C–D, each column represents one individual. Rows represent each of the potentially methylated 26 CpG sites in the rDNA promoter in 5′ to 3′ orientation. The data (% methylation for a given CpG) are from analyzing 20 unique clones for each subject.

To determine the correlations between AD pathology and methyl-cytosine (mC) content in the rDNA promoter, genomic DNA was prepared from post mortem cerebrocortical samples of 23 AD patients, 10 MCI patients and 24 age-matched control individuals (Table 1). The mC analysis consisted of bisulfite-mediated non-methyl-cytosine conversion (C to T) followed by PCR amplification of the rDNA promoter fragment (Fig. 1A–B). After cloning of the PCR product, rDNA promoter sequence was analyzed in individual clones. Because rDNA is composed of the repeated 45S rRNA genes, at least 20 unique clones were analyzed for each subject. The heat maps in Fig. 1C–D illustrate average mCpG fractions for each rDNA CpG site in each of the analyzed subjects. Importantly, sequence analysis of the unconverted genomic DNA from all AD- and control subjects revealed no CpG polymorphisms in the rDNA promoter (data not shown). Although potential polymorphisms removing CpGs were detected in individual rDNA clones, their relative rarity and limited extent (of 1400 analyzed clones, 3 showed loss of one or two CpG sites/clone) further support the notion that bisulfite mapping analysis was not confounded by variations in DNA sequence. Finally, at least for the CpG site #23 (position −9 relative to transcription start site) we validated observations obtained with bisulfite mapping using the methylation-sensitive restriction endonuclease HpaII (Figure S1).

**Table 1:** Demographic and psychiatric profile of AD patients and controls.

	Control	MCI	AD
**Number of subjects**	24	10	23
**Age (years)**	85.46±1.25^a^	86.6±1.86^a^	83.5±1.24^a^
**Sex (F∶M)**	16∶8	7∶3	12∶11
**Brain weight (g)**	1176±25.27^a^	1150±30.48^a^	1096±27.31^a^
**PMI (hr)** b	2.64±0.15^a^	3.05±0.36^a^	3.03±0.10^a^
**MMSE** c	28.37±0.33^a^	22.33±1.84^a^	9.82±1.34^a^

**Footnotes:**

a Mean Standard Error of the Mean (SEM);

b PMI, *Post Mortem* Interval;

c MMSE, Minimental State Examination score.

**Figure 2:**
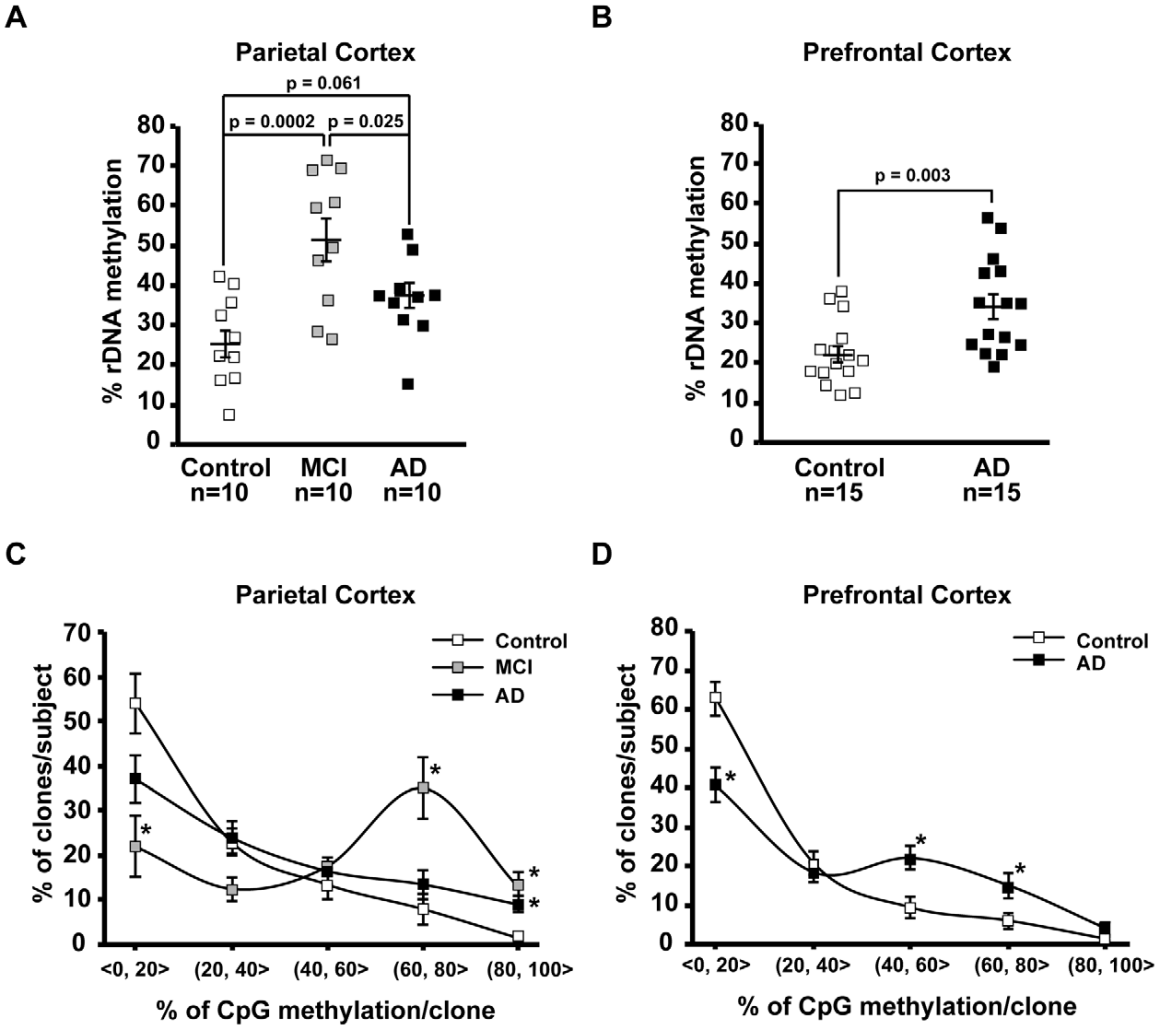
Effects of MCI and AD on mC content in the rDNA promoter. ***A–B***, Average % mC in the rDNA promoter (% rDNA methylation) in the parietal- (A), and the prefrontal cortex (B). Individual values are depicted by squares; mean values are indicated by the lines intersecting the error bars (SEM); p values and number of analyzed cases (n) are indicated. In the AD-affected prefrontal cortex, the rDNA promoter is hypermethylated. ***C–D***, The distribution of differentially methylated rDNA promoter clones is affected by MCI and AD. For each sample, % rDNA clones were determined within 5 ranges of mC content; a range definition “(20,40>” means more than 20 and less than-, or, equal to 40% mC. Mean values ±SEM are presented for each group of subjects. The bias towards hypomethylated clones is present in controls (ANOVA, p<0.001). That trend is disrupted or flattened in MCI or AD, respectively (two-way ANOVA, interaction between mC range and diagnosis, p<0.001); *, p<0.05 for post-hoc comparisons against in-range controls.

As in the human rDNA promoter average density of CpG methylation across all individual CpG sites appears critical for silencing nucleolar chromatin [7], we initially compared that parameter between the studied groups (Fig. 2). In the parietal cortex samples from control individuals, the mean mCpG density ± SEM in the rDNA promoter was 25.35±3.55% (Fig. 2A). In the MCI-affected prefrontal cortex, that number was 50.67±5.33% (control vs. MCI, p = 0.0002, Fig. 2A). Although the mCpG content decreased in the AD parietal cortex to 36.79±3.21% (AD vs. MCI, p = 0.025, Fig. 2A), the hypermethylation trend was still present in that region (AD vs. control, p = 0.061, Fig. 2A). In the prefrontal cortex, mCpG content in the rDNA promoter increased from 22.2±2.08% in controls to 34±3.02% in AD (p = 0.003, Fig. 2B). The AD-associated changes of the mCpG density in the rDNA promoter were accompanied by altered distribution of differentially methylated rDNA promoter units. In average control parietal cortex, 77% and 9.5% of all analyzed clones contained ≤40 and >60% mCpGs, respectively (Fig. 2C, p<0.001). In addition, 54% clones contained 20% or less mCpGs (Fig. 2C). Similar distribution pattern was also observed in the control prefrontal region indicating that in human cerebral cortex most rDNA units remain relatively unmethylated. In contrast, that pattern was reversed in the MCI parietal cortex (34.5 and 48% clones with ≤40 and >60% mCpGs, respectively, Fig. 2C) or flattened in the AD parietal and the prefrontal cortices (Fig. 2C–D). Such flattening appeared greater in the prefrontal than the parietal cortex (Fig. 2C–D).

In the AD cerebellum, which lacks advanced AD-type pathology, mCpG frequency in the rDNA promoter was similar in AD and control groups (20.8±2.8% vs. 28±5.7%, respectively, ANOVA p>0.05, Fig. S2). In addition, analysis of total mC content in the genomic DNA from the parietal- and the prefrontal cortex or the cerebellum revealed no differences between AD, MCI, and control individuals (Fig. S3). Hence, in tissue with AD-induced neurodegeneration, the rDNA promoter but not the whole genome becomes hypermethylated. In addition, the greatest rDNA hypermethylation is associated with relatively early stages of AD pathology.

### Distribution of the AD-hypermethylated CpGs in the rDNA promoter

**Figure 3:**
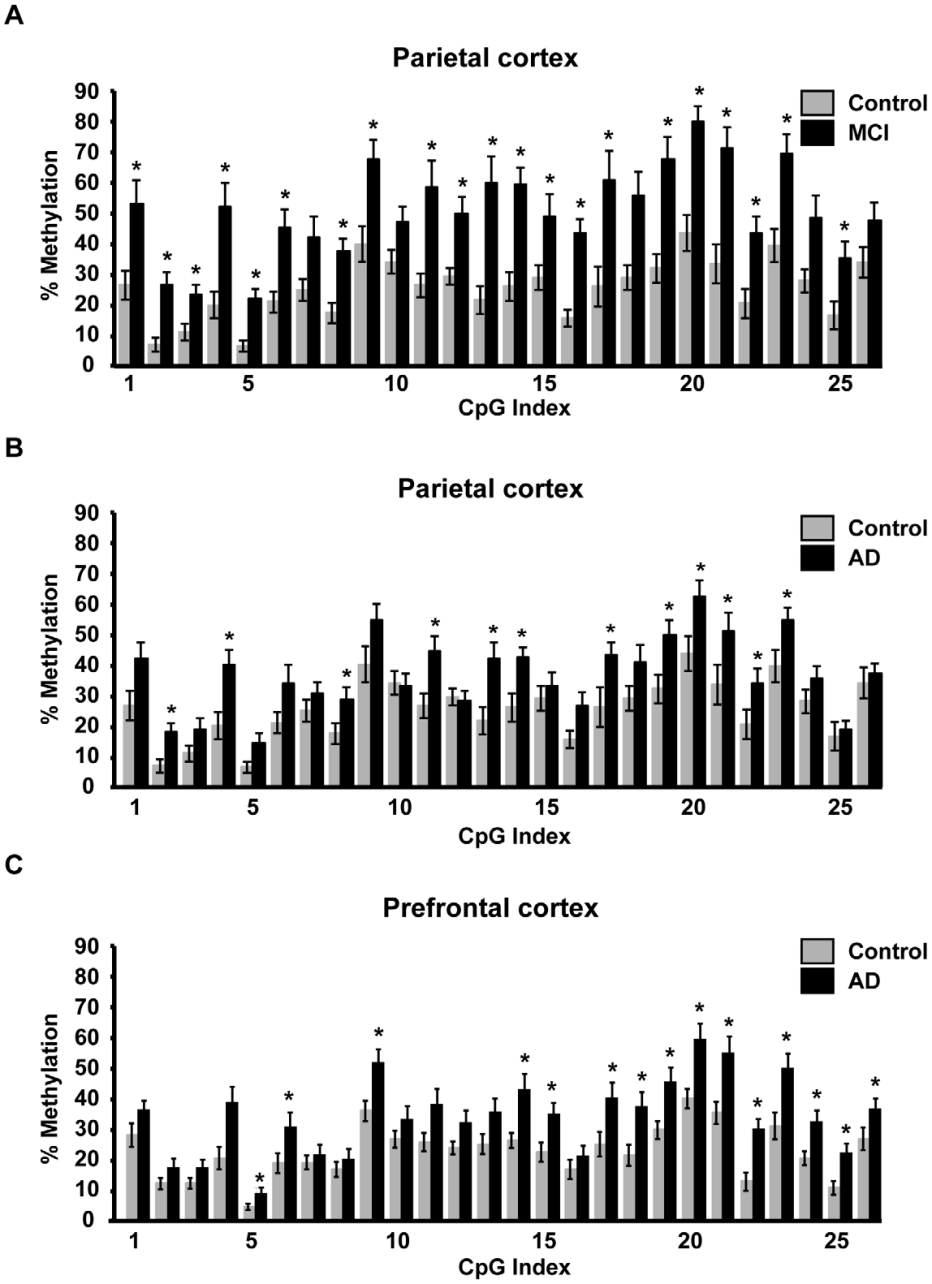
Effects of MCI and AD on distribution of mCpGs across the rDNA promoter. Percent CpG methylation at each of 26 individual CpG sites located in the rDNA promoter in samples from parietal- (**A–B**) and prefrontal cortex (**C**). For the purpose of clarity, parietal cortex data for MCI and AD groups are presented on separate graphs. The controls are the same in both cases. Data are means ± SEM for each analyzed group of cases (n as in Fig. 1); *, p<0.05 (SAM statistics as compared to controls).

**Figure 4:**
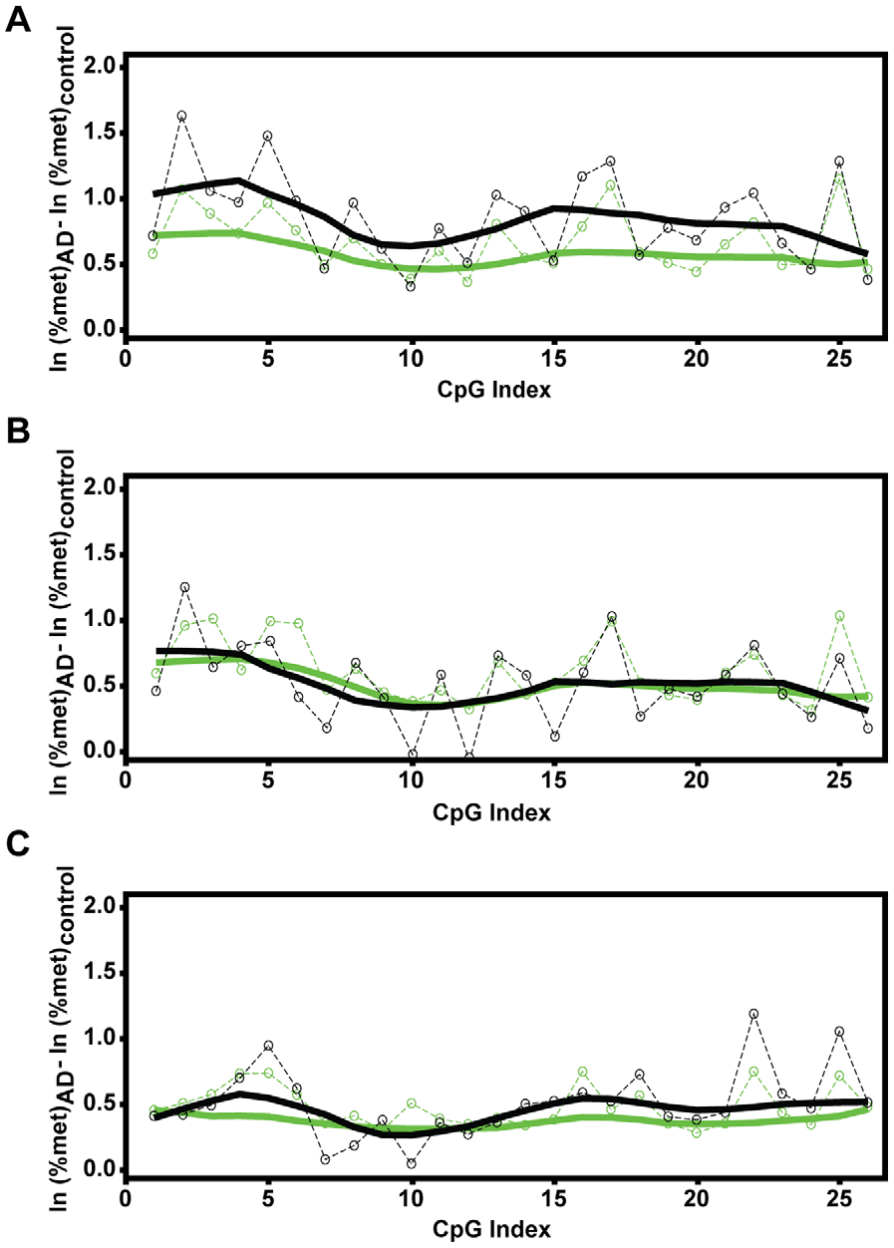
Local regression analysis of mC content across the rDNA promoter. After logarithmic transformation of the data from Fig. 3, the mean differences in methylation of each of 26 individual CpG sites in the rDNA promoter were determined as indicated. ***A***, Local regression analysis of the mean difference between MCI- and control groups (parietal cortex) indicates significant hypermethylation of all CpGs of the rDNA promoter. The circles indicate the actual mean difference values; the black solid line represents a smoothed regression fit of those values across the rDNA promoter region; the green lines indicate the border of the 95% confidence range for the actual values (dashed line) or the smoothed regression (solid line). Note that the black smoothed regression line does not leave the 95% confidence range, indicating a significant hypermethylation trend for all the CpG sites. ***B–C***, Similar analysis of the data for the AD samples from the parietal (B) and the prefrontal cortex (C) identifies AD-associated hypermethylation of large portions of the rDNA promoter.

Comparison of 26 individual CpGs that are located in the rDNA promoter revealed that 21 of them are significantly hypermethylated in MCI parietal cortex (SAM p<0.05, Fig. 3A). In the upstream control element (UCE; positions −186 to −51 relative to the transcription start site) 15 of 18 CpGs were hypermethylated. In the core promoter (CP; positions −49 to 20), increased methylation was observed in 6 of 8 CpGs. In AD parietal cortex, the number of hypermethylated CpGs was reduced to 12 (7 in the UCE and 5 in the CP, SAM p<0.05, Fig. 3B). In AD prefrontal cortex, there were 7 and 8 hypermethylated CpGs in the UCE and the CP, respectively (SAM p<0.05, Fig. 3C). In addition, in all groups many “unaffected” CpGs showed MCI- and/or AD-associated hypermethylation trends resulting in apparently even hypermethylation pattern across large portions of the rDNA promoter (Fig. 3). Indeed, local regression analysis, which took such trends into account, revealed that hypermethylation is a significant and uniform direction of the MCI effects on all CpGs of the rDNA promoter (Fig. 4A). Likewise, extended hypermethylation areas of the rDNA promoter were revealed in the cerebro-cortical samples from the AD group (Fig. 4B–C). MCI/AD-associated hypermethylation of large portions of the rDNA promoter is consistent with appearance of silenced nucleolar chromatin, which in humans is linked to an overall increase in methylation density of the rDNA promoter [7]. In addition, robust expression of rDNA hypermethylation in MCI suggests that such a nucleolar silencing appears at relatively early stages of AD pathology.

At the single CpG level, no AD hypermethylation was observed in the cerebellar rDNA promoter (Fig. S4). While some CpGs showed a trend towards hypomethylation, local regression analysis did not confirm significance of such changes (Fig. S4). These findings support specificity of the association between hypermethylation of the rDNA promoter and AD-related neurodegeneration in the cerebral cortex.

### Biological significance of the AD-associated hypermethylation of the rDNA promoter

The rDNA promoter methylation reduces Pol1-mediated transcription of rRNA [7]. In post mortem material, high sensitivity of Pol1 to hypoxia limits the use of direct indicators of Pol1 activity including expression of the relatively unstable 45S rRNA primary transcript [20]. Conversely, very high stability of mature rRNAs makes them poor Pol1 indicators as well [18]. Consistent with that notion, the only significant change in rRNA levels was reduction of 28S rRNA in AD prefrontal cortex (0.35±0.09 fold controls, p = 0.0083), while 18S rRNA and 5.8 rRNA were unaffected (Fig. S5).

Another consequence of rDNA promoter methylation is increased stability of rDNA repeats presumably due to decreased recombination rate between them [10]. Since rDNA hypomethylation reduced rDNA content in a cell line and declining rDNA levels were reported during human aging [10], [21], one could expect that AD-associated rDNA hypermethylation may result in higher genomic content of rDNA as compared to age-matched controls.

**Figure 5:**
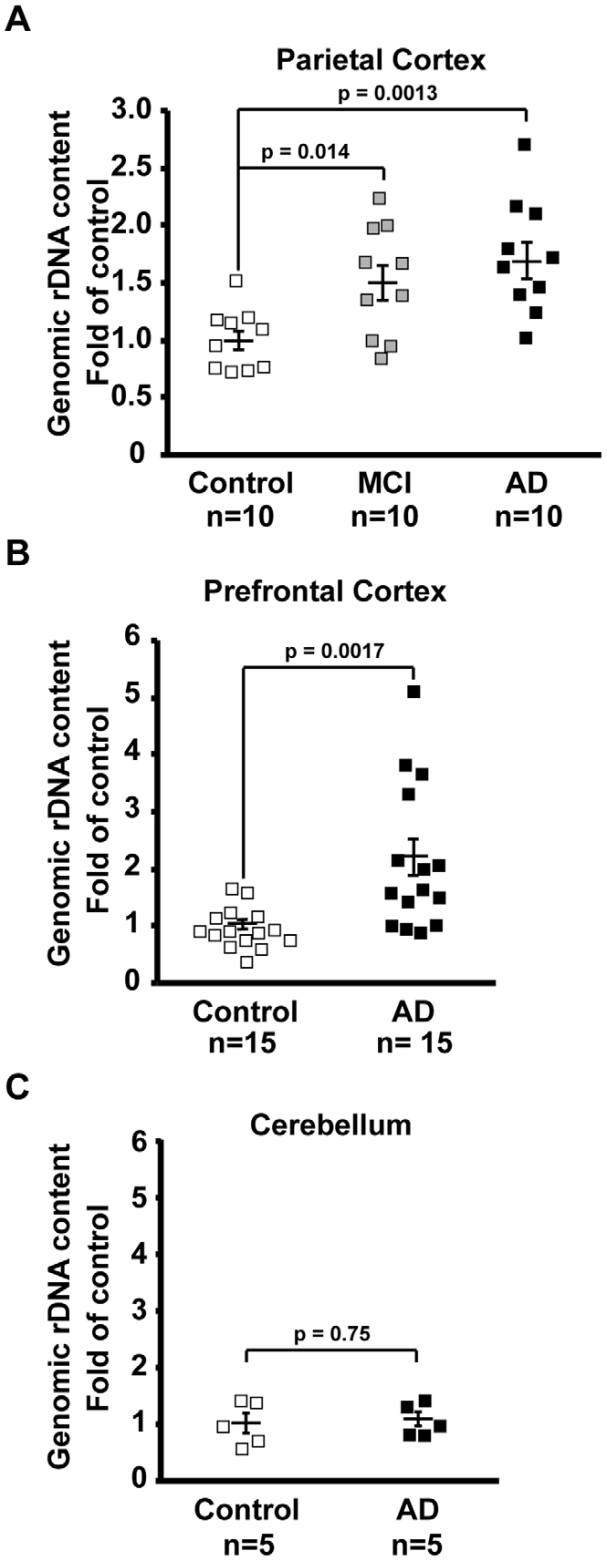
Increased genomic rDNA content in cerebro-cortical samples from MCI- and AD individuals. Genomic rDNA was quantified by qRT-PCR; for normalization, *tRNA-K-CTT* gene was selected as its unclustered copies are scattered throughout several human chromosomes providing a good control of the non-rDNA genome stability. The relative rDNA content was calculated as a ratio of rDNA to tRNA-K-CTT. ***A***, In parietal cortex increased rDNA content was observed both in MCI and AD. ***B–C***, AD- associated increase in rDNA content in the prefrontal cortex (B) but not the cerebellum (C). Individual values are depicted by squares; mean values are indicated by the lines intersecting the error bars (SEM); p values and numbers of analyzed cases (n) are shown.

To determine rDNA content a quantitative real time-PCR (qRT-PCR) method that was originally developed to analyze *D. melanogaster* rDNA was adapted for human samples [22]. Amplification of rDNA was achieved by primers complementary to the 18S rRNA exon. For normalization, the gene for *tRNA^K-CTT^* was selected as it represents repeated DNA (17 almost identical copies per haploid) that, in contrast to rDNA, is not clustered together [23]. Therefore, *tRNA^K-CTT^* gene provides insight into genomic stability across multiple chromosomes while being less likely than rDNA to undergo recombination-associated loss. In the MCI and AD parietal cortex, the mean rDNA/*tRNAK-CTT* ratio was 1.5- and 1.69- fold higher than in age-matched controls, respectively (MCI vs. control, p = 0.0014; AD vs. control, p = 0.0013, Fig. 5A). Similar increase was also observed in AD prefrontal cortex (2.17 fold controls, p = 0.0017, Fig. 5B). In the cerebellum, similar values of rDNA/*tRNAK-CTT* ratio were observed in AD and control groups (p>0.05, Fig. 5C). As for all analyzed samples the velocities of rDNA- and *tRNAK-CTT* PCR product accumulation over the subsequent qRT-PCR cycles were similar irrespective of diagnosis or the rDNA methylation, we concluded that the identified differences in rDNA content were not artifacts of differential amplification efficiency. Thus, in AD-related neurodegeneration, hypermethylation of the rDNA promoter may trigger functional changes of nucleolar chromatin including increased rDNA stability.

## Discussion

This study demonstrated hypermethylation of the rDNA promoter correlated with AD-type pathology in the parietal and prefrontal cortex but not the cerebellum. The level and range of the hypermethylation was relatively greater in MCI than in late stage AD. There was a MCI-associated reversal of a bias towards enrichment for hypomethylated rDNA units that was present in controls as well as the late stage AD. In MCI the increased methylation density appeared uniformly distributed across the rDNA promoter. However, in later stage AD, large portions of the rDNA promoter remained hypermethylated. In addition, the MCI/AD-associated hypermethylation of the rDNA promoter was accompanied by increased content of rDNA suggesting its greater stability. Conversely, global genomic content of mC was unrelated to AD status. Thus, in cerebral cortex, AD-related hypermethylation of the rDNA promoter may be associated with repressive chromatin conformation in the nucleolus likely contributing to diminished ribosomal biogenesis and reduced protein synthesis.

The excessive silencing of rDNA is the most robust change in the AD brain epigenome that has so far been identified. Unlike other known epigenetic irregularities in AD, hypermethylation of the rDNA promoter appears to be a component of an AD associated pathological cascade. While similar rDNA mC content has been reported in peripheral blood cells from AD- and control subjects [24], our data suggest that changes in rDNA promoter methylation are specific to cells directly confronted with AD pathology.

Our results demonstrate that rDNA hypermethylation is most strongly associated with early stages of AD pathology. Such kinetics could indicate that the primary site of rDNA hypermethylation are nuclei of degenerating neurons that are lost in the later stage AD. Conversely, it is tempting to speculate that increasing reactive gliosis could be associated with reappearance of the active, hypomethylated rDNA units. However, we can not exclude an alternative possibility that MCI/AD-associated hypermethylation of rDNA is an effect of increased frequency of non-neuronal cells such as reactive glia if their rDNA methylation levels are higher than those of normal resident cells of the analyzed regions.

In cerebral cortex, the effects of AD on rDNA methylation were not accompanied by reduced levels of mature rRNAs except a decline of 28S rRNA in prefrontal cortex. Similar decline of 28S but not 5.8S or 18S rRNA was reported by Ding et al. in parietal cortex from MCI- and AD subjects [12]. However, we observed no 28S reduction in that region. As we used similar methodology to analyze samples from the same cohort as Ding et al., this discrepancy may reflect high individual and/or regional variability of rRNA levels. The concept that reduced rRNA transcription results primarily in qualitative decline of the existing ribosomal pool is supported by the observations that (*i*), at least upon inhibition of Pol1 activity, brain rRNA is relatively very stable [18], and, (*ii*) oxidized rRNA species accumulate in AD-affected brain structures [11], [12], [13]. Therefore, a decrease in nucleolar transcription, which is an expected consequence of the AD-related rDNA hypermethylation, may have relatively greater negative effect on rRNA quality but not its quantity.

Data presented in this report indicate that the AD-associated hypermethylation of the rDNA promoter is linked to increased stability of the rDNA loci. These observations are in agreement with a recent report that in a mouse cell line, rDNA content has declined following knockdown of the NoRC component, the TTF-1-interacting protein-5 (TIP5) that is critical for DNA methyltransferase recruitment to the rDNA promoter [10]. Conversely, TIP5 depletion or overexpression increased or decreased rRNA transcription/cell growth, respectively [9], [10]. Hence, despite increasing rDNA content, the net effect of the rDNA promoter hypermethylation on ribosomal biogenesis is negative.

The notion that hypermethylation-induced chromatin silencing stabilizes rDNA preventing its loss during aging is consistent with the established anti-aging effects of the yeast chromatin silencer sirtuin, which has been proposed to decelerate yeast cell aging by blocking rDNA recombination [25]. Although initial human studies with limited number of subjects produced conflicting results on rDNA maintenance in aging [26], [27], recent analysis of a larger cohort documented age-associated loss of rDNA in adipose tissue [21]. If AD-associated rDNA hypermethylation prevents rDNA loss, why is rDNA content elevated not only in MCI but also in advanced AD when rDNA hypermethylation is diminished? Such a discrepancy can be explained by a model in which the reduction in rDNA methylation in late AD is associated with increasing content of reactive glia. As dividing cells, reactive glia are expected to have no age-related rDNA loss [27]. In contrast, in MCI, increased rDNA content will be mostly from degenerating neurons which would also have their rDNA hypermethylated. In support of such scenario, rDNA hypermethylation showed strong correlation with rDNA content in MCI (R^2^ = 0.73, p = 0.002) but not in control- or AD groups. Overall, data presented in the current report suggest that rDNA loss may occur in aging human cerebral cortex and that this process could be antagonized by methylation of the rDNA promoter.

Given the fact that rDNA consist of hundreds of repeated rRNA genes, the extensive hypermethylation of the rDNA promoter is the greatest AD-related abnormality in the epigenetic landscape of the genome that has been so far identified. As hypermethylation of rDNA has been associated with aging, the AD effects on rDNA methylation may represent an excessive aging response [28], [29], [30]. Interestingly, the other identified DNA methylation irregularities in AD brains have been also interpreted as enhancement of epigenetic changes that occur during physiological aging [5], [6].

Currently, it is unknown what is a direct trigger for such an exacerbated “aging” of the brain rDNA in AD. However, it is tempting to speculate that it is a consequence of exposure(s) to such socio-environmental factors facilitating AD development as social stress, neurotrauma or heavy metals [4]. Alternatively, nucleolar silencing could be a secondary response to AD-associated β-amyloidosis, tauopathy, inflammation and/or oxidative stress. Finally, it may represent an action of a novel genetic factor that by increasing rDNA methylation and/or disrupting its demethylation increases AD susceptibility. For instance, a DNA demethylation enzyme TET1 is located in a chromosome 10 region that harbors a relatively strong susceptibility locus for the late onset AD [31], [32]. Our future studies will address the possible causes of AD-associated changes in rDNA methylation.

Taken together, our results indicate that hypermethylation of the rDNA promoter is a likely contributor to ribosomal deficiency in AD-affected brains. By reducing nucleolar transcription and subsequently decelerating ribosomal turnover, hypermethylation of the rDNA promoter may participate in qualitative decline of the rRNA component of brain ribosomes contributing to AD-associated synapse loss and dementia. Therefore, the epigenetic regulation of nucleolar transcription is a potential target to intervene against AD. Finally, nucleolar silencing may contribute to other age-related neurodegenerative diseases.

## Materials and Methods

### Subjects and sample preparation

The donors were participants of the IRB-approved University of Kentucky AD Center cohort and were followed for at least 2 years before death [33]. The follow up included annual Minimental State Examination (MMSE) as well as neurological and physical examinations. The donors had no history of substance abuse, head injury, encephalitis, meningitis, epilepsy, or stroke/transient ischemic attack. The MMSE score closest to death was used as an indicator of overall cognitive status. During autopsy (5 or less hours after death), tissue samples including parietal cortex (Brodmann Areas 39 and 40), prefrontal cortex (Brodmann Area 9) and cerebellum were processed for neuropathological evaluations or flash-frozen in liquid nitrogen and stored at −80°C, as described previously [33], [34]. All included AD subjects met the clinical and histophathological criteria for diagnosis of AD [33], [34]. The MCI group was categorized as defined previously according to conventional clinical-pathological consensus criteria [33], [34]. Summary of donor data is presented in Table 1.

### Genotyping of the rDNA promoter region

Genomic DNA was isolated from flash frozen tissue samples using DNeasy Blood & Tissue Kit (Qiagen). A fragment including the rDNA promoter region was PCR-amplified and sequenced bidirectionally (detailed primer information is in Table S1).

### Bisulfite Mapping of rDNA Methylation

A previously described methodology was used with modifications [8]. Briefly, genomic DNA was isolated from frozen samples and unmethylated cytosines were converted with bisulfite using EZ DNA Methylation-Direct Kit (Zymo Research). The converted DNA was a template for PCR amplification of the rDNA promoter containing 26 potential mCpGs (detailed primer information is in Table S1). The PCR product was cloned in pGEM-T vector and at least 20 clones from each sample were sequenced using the SP6 universal reverse primer. Sequencing results were analyzed with software developed in our laboratory that can be obtained upon request. Clones with incomplete bisulfite conversion of non-CpG cytosines were excluded from analysis. To avoid a bias due to multiplication of identical clones, only such clones that represented unique patterns of CpG methylation were included in the final analysis of each sample.

### Methyl-sensitive restriction enzyme analysis of methylation

Methylated-C sensitive restriction analysis was performed as described previously [35]. DNA (200 ng) was treated with HpaII over night and qRT PCR was run with primers specific to rDNA promoter fragment that contain the CpG#23 (position −9 from the transcription start site, Fig. 1B; primer details are in Table S1). Results were normalized for undigested (input) DNA. The qRT-PCR data were analyzed using the standard curve method.

### Methylated DNA content in the genome

Genomic DNA was isolated as for genotyping. The content of mC was determined using 100 ng of DNA/sample and the commercial mC ELISA system (Imprint Methylated DNA Quantification Kit, Sigma-Aldrich). For each sample, triplicate determinations were performed.

### Analysis of genomic rDNA content

The previously described qRT-PCR technique was used to determine rDNA content in the genomic DNA [22]. The amplified portions of the rDNA cistron mapped within the 18S rRNA coding region. The content of *tRNA^K-CTT^* gene DNA was used as a normalization control. Details concerning the primers are in Table S1. The qRT-PCR data were analyzed using the standard curve method.

### Analysis of 18S, 5.8S and 28S rRNA levels

RNA isolation, reverse-transcription with random hexamers and quantitative real time PCR (qRT-PCR) with SYBR Green (SuperArray Bioscience) were performed using standard methodology that was described before [36] except that qRT-PCR data were analyzed using the standard curve method. Detailed primer information is in Table S1.

### Statistical analysis

Comparisons of individual rDNA methylation sites were accomplished using a modified significance analysis of microarrays (SAM) [37]. The detailed description of the modifications including addition of local regression analysis is provided as a supplementary information (Methods S1). SAM has been successfully applied to analyze complex genome methylation patterns such as those in rDNA [38]. Importantly, the modified SAM model makes no functional assumptions about the methylation data at different sites and takes proper account of the correlation structure between sites at the same time allowing for the control of the probability of false discoveries. The average rDNA- and total genomic DNA mC levels as well as rDNA content were compared by ANOVA and Fisher LSD *post-hoc* tests.

## Supporting Information

Figure S1 **Confirmation of rDNA promoter hypermethylation in MCI parietal cortex using the mC-sensitive restriction endonuclease HpaII.** ***A***, Hypermethylation of rDNA promoter CpG#23 (position -9 relative to transcription start site) was confirmed by a mC-sensitive restriction endonuclease HpaII. If CpG#23 was unmethylated, cleavage by HpaII destroyed a template for qRT PCR. Increased levels of the HpaII-resistant template indicated rise in mC frequency at that site. The results for HpaII-treated genomic DNA were normalized against the non-treated DNA. Data are means ± SEM from 5 MCI- and 5 control individuals. ***B***, Hypermethylation of CpG#23 as observed in the same individuals using bisulfite sequencing (for more details, see Figs. 1&3).
(TIF)

Figure S2 **Effects of AD on mC content of the rDNA promoter in the AD pathology-free cerebellum.** ***A***, Average % mC in the rDNA promoter (% rDNA methylation). Individual values are depicted by squares; mean values are indicated by the lines intersecting the error bars (SEM); p values and numbers of analyzed cases (n) are indicated. In the AD-affected prefrontal cortex, the rDNA promoter is hypermethylated. ***B***, The distribution of differentially methylated rDNA promoter clones indicates overrepresentation of hypomethylated rDNA units in control samples (p<0.001). Similar pattern is present in AD. For more details, see description of the Fig. 2.
(TIF)

Figure S3 **Effects of MCI and AD on mC content in total genomic DNA as determined with an mC-specific ELISA.** ***A***, In parietal cortex, neither MCI nor AD affected mC content in total genomic DNA (global DNA methylation). ***B–C***, AD had also no effects on that parameter in the prefrontal cortex (B) or the cerebellum (C). Individual values are depicted by squares; mean values are indicated by the lines intersecting the error bars (SEM); p values and numbers of analyzed cases (n) are shown.
(TIF)

Figure S4 **Effect of AD on distribution of CpG methylation across the rDNA promoter in the cerebellum.** ***A***, Percent methylation at each of 26 individual CpG sites located in the rDNA promoter region. The data represent averages ± SEM from 5 AD- and 5 non-AD control individuals; *, p<0.05 (SAM statistics). ***B***, Local regression analysis of the AD effects on rDNA promoter methylation in the cerebellum. Mean differences between AD- and control groups are plotted. The circles indicate the actual mean difference values; the black solid line represents a smoothed regression fit of those values across the rDNA promoter region; the green lines indicate the border of the 95% confidence range for the actual values (dashed line) or the smoothed regression (solid line). For more details, see description of Figs. 3–4.
(TIF)

Figure S5 **Effects of MCI and AD on 18S-, 5.8S- and 28S rRNA levels.** Quantitative real time PCR analysis of rRNAs was performed using the standard curve method. ***A–C***, In parietal cortex, MCI or AD did not affect 18S-, 5.8S-, or 28S rRNA levels. ***D–F***, In prefrontal cortex an AD- associated decrease of 28S-, but not, 18S-, or 5.8S rRNA was observed. Individual values are depicted by squares; mean values are indicated by the horizontal bars that intersect the error bars (SEM); p values are shown; n = 10 or 15 for each group in A–C or D–F, respectively.
(TIF)

Table S1 **Primers and PCR conditions.**
(DOC)

Methods S1 **A text file containing detailed description of the modified SAM statistics.**
(DOC)
