# Supplementary figures and images for: Epigenetic Silencing of Nucleolar rRNA Genes in Alzheimer's Disease
Source: PLoS One. 2011 Jul 22;6(7):e22585. doi: 10.1371/journal.pone.0022585 (PMC3142181; doi:10.1371/journal.pone.0022585)

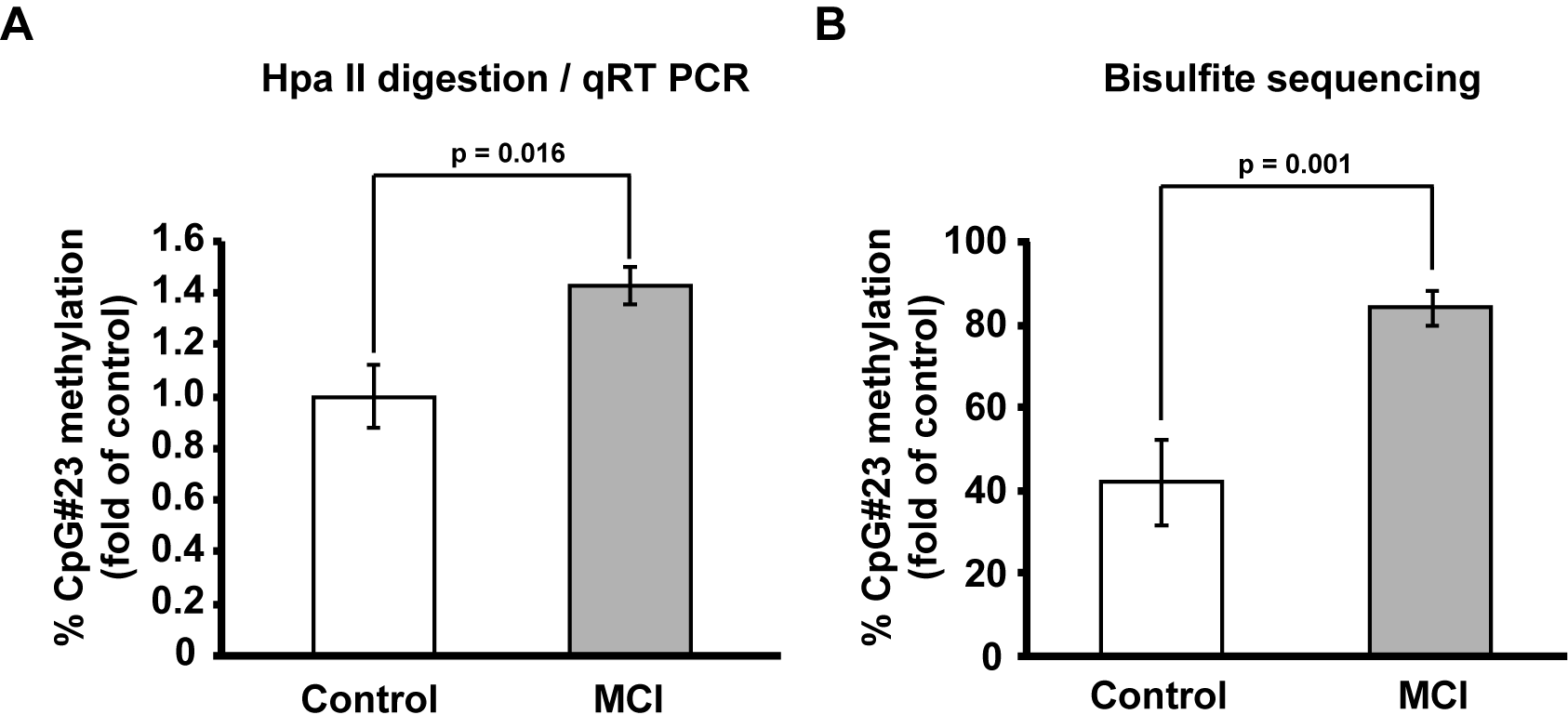

Supplement: Figure S1 — Confirmation of rDNA promoter hypermethylation in MCI parietal cortex using the mC-sensitive restriction endonuclease HpaII. A, Hypermethylation of rDNA promoter CpG#23 (position -9 relative to transcription start site) was confirmed by a mC-sensitive restriction endonuclease HpaII. If CpG#23 was unmethylated, cleavage by HpaII destroyed a template for qRT PCR. Increased levels of the HpaII-resistant template indicated rise in mC frequency at that site. The results for HpaII-treated genomic DNA were normalized against the non-treated DNA. Data are means ± SEM from 5 MCI- and 5 control individuals. B, Hypermethylation of CpG#23 as observed in the same individuals using bisulfite sequencing (for more details, see Figs. 1&3). (TIF) [file pone.0022585.s001.tif]

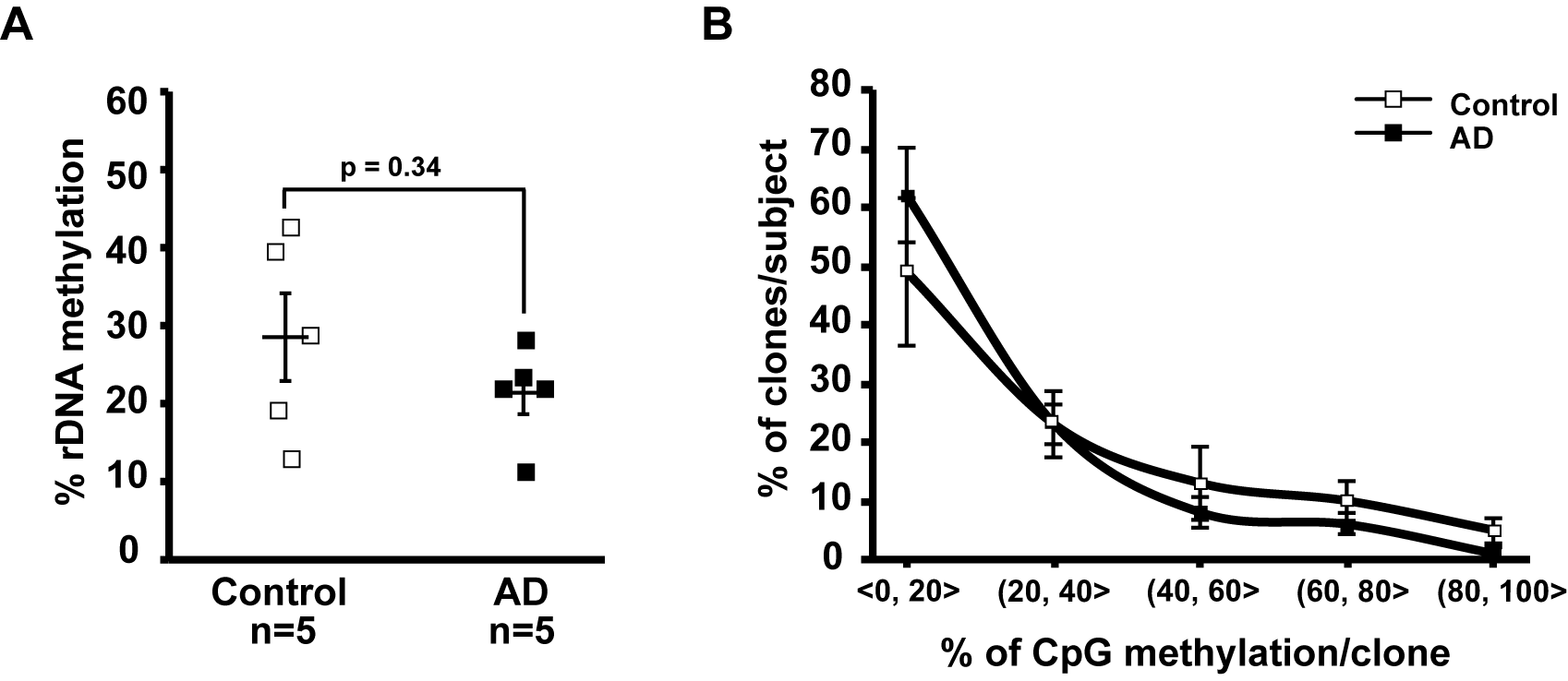

Supplement: Figure S2 — Effects of AD on mC content of the rDNA promoter in the AD pathology-free cerebellum. A, Average % mC in the rDNA promoter (% rDNA methylation). Individual values are depicted by squares; mean values are indicated by the lines intersecting the error bars (SEM); p values and numbers of analyzed cases (n) are indicated. In the AD-affected prefrontal cortex, the rDNA promoter is hypermethylated. B, The distribution of differentially methylated rDNA promoter clones indicates overrepresentation of hypomethylated rDNA units in control samples (p<0.001). Similar pattern is present in AD. For more details, see description of the Fig. 2. (TIF) [file pone.0022585.s002.tif]

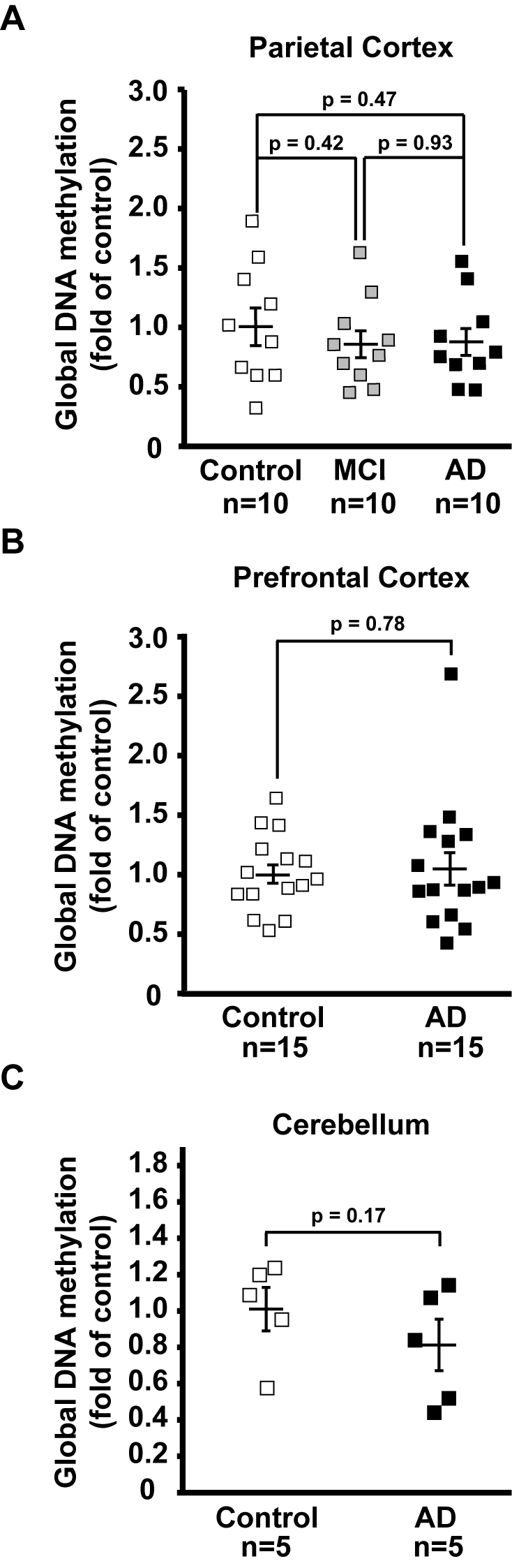

Supplement: Figure S3 — Effects of MCI and AD on mC content in total genomic DNA as determined with an mC-specific ELISA. A, In parietal cortex, neither MCI nor AD affected mC content in total genomic DNA (global DNA methylation). B–C, AD had also no effects on that parameter in the prefrontal cortex (B) or the cerebellum (C). Individual values are depicted by squares; mean values are indicated by the lines intersecting the error bars (SEM); p values and numbers of analyzed cases (n) are shown. (TIF) [file pone.0022585.s003.tif]

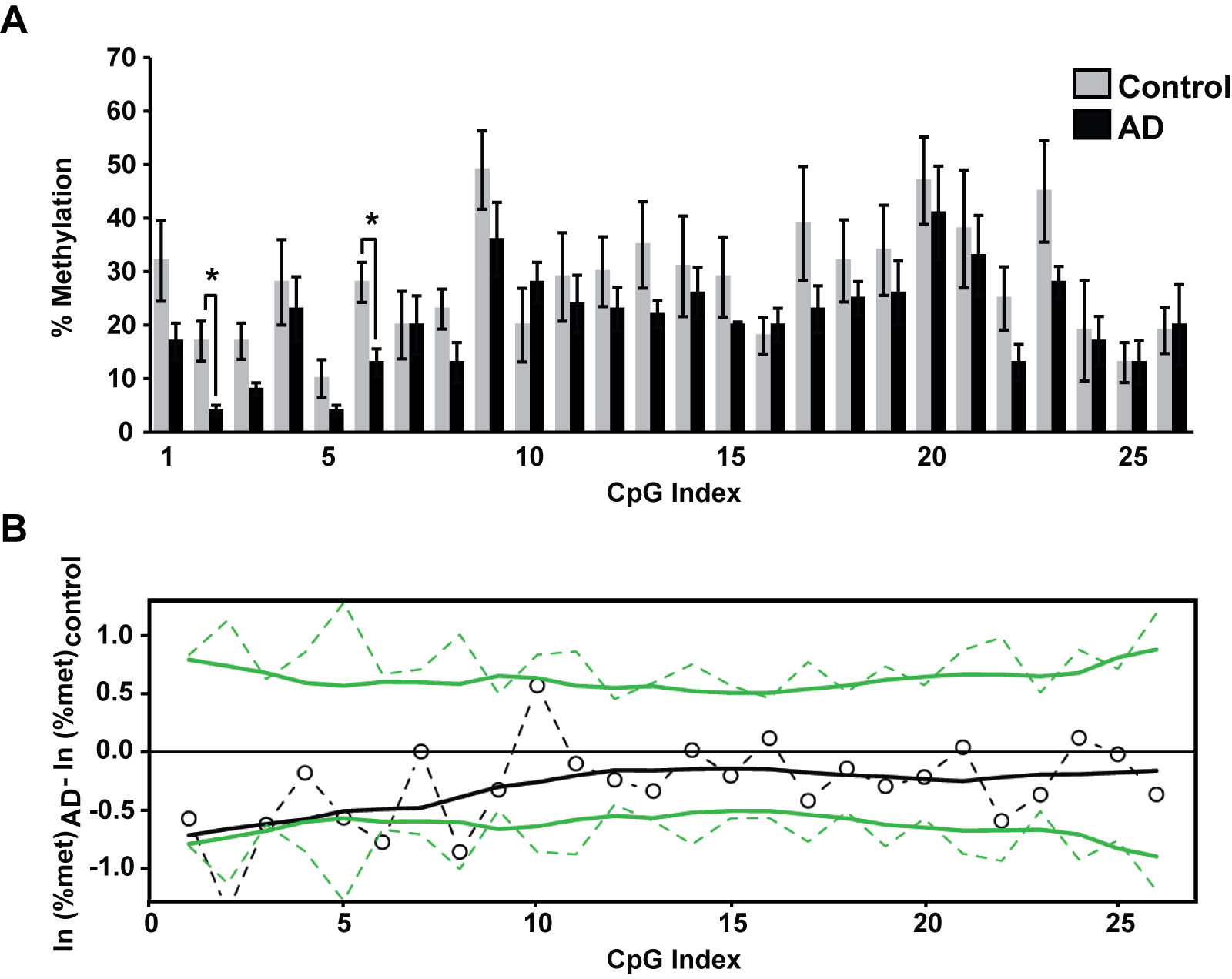

Supplement: Figure S4 — Effect of AD on distribution of CpG methylation across the rDNA promoter in the cerebellum. A, Percent methylation at each of 26 individual CpG sites located in the rDNA promoter region. The data represent averages ± SEM from 5 AD- and 5 non-AD control individuals; *, p<0.05 (SAM statistics). B, Local regression analysis of the AD effects on rDNA promoter methylation in the cerebellum. Mean differences between AD- and control groups are plotted. The circles indicate the actual mean difference values; the black solid line represents a smoothed regression fit of those values across the rDNA promoter region; the green lines indicate the border of the 95% confidence range for the actual values (dashed line) or the smoothed regression (solid line). For more details, see description of Figs. 3–4. (TIF) [file pone.0022585.s004.tif]

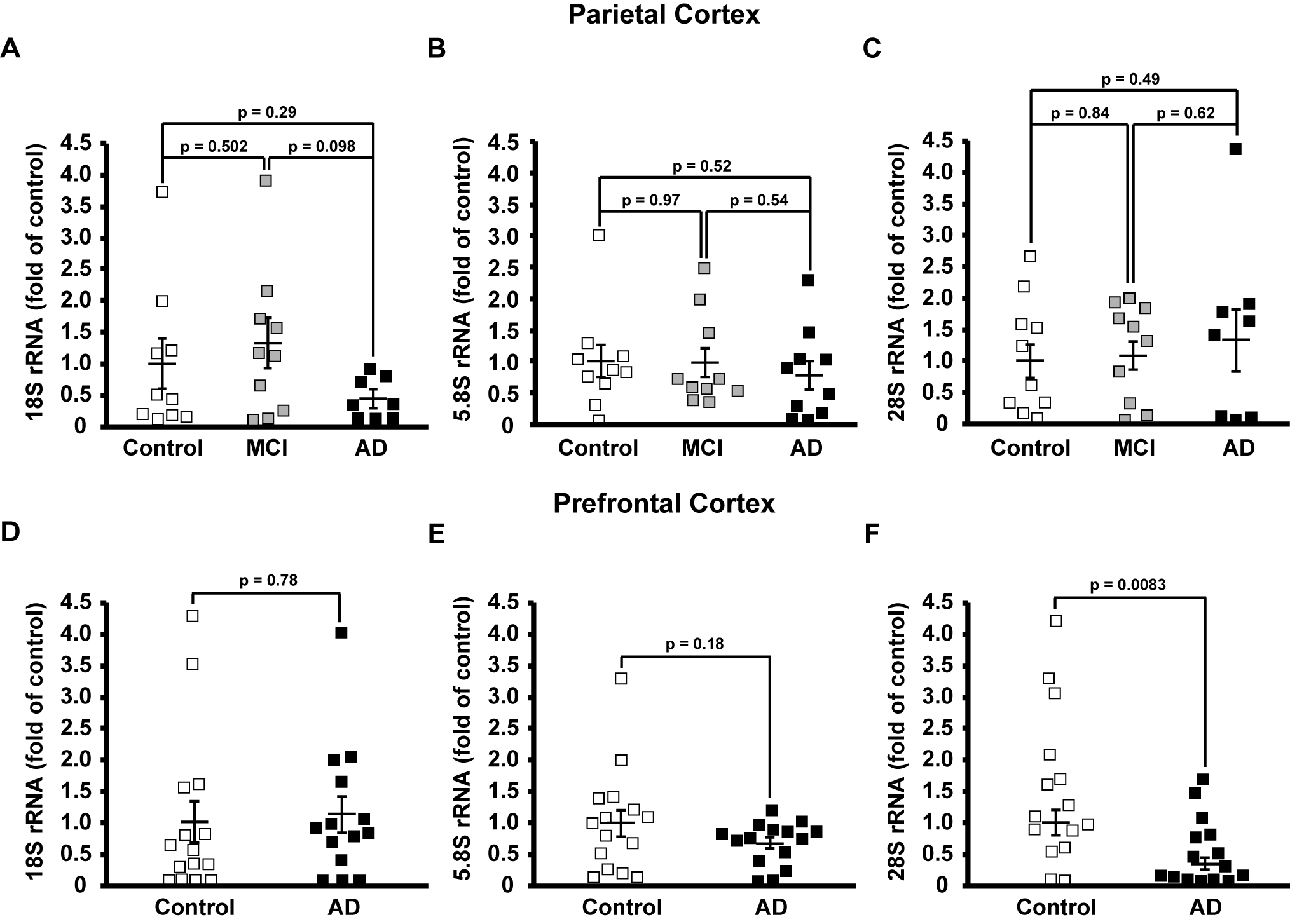

Supplement: Figure S5 — Effects of MCI and AD on 18S-, 5.8S- and 28S rRNA levels. Quantitative real time PCR analysis of rRNAs was performed using the standard curve method. A–C, In parietal cortex, MCI or AD did not affect 18S-, 5.8S-, or 28S rRNA levels. D–F, In prefrontal cortex an AD- associated decrease of 28S-, but not, 18S-, or 5.8S rRNA was observed. Individual values are depicted by squares; mean values are indicated by the horizontal bars that intersect the error bars (SEM); p values are shown; n = 10 or 15 for each group in A–C or D–F, respectively. (TIF) [file pone.0022585.s005.tif]
