# Supplementary material for: Epigenetic Silencing of Nucleolar rRNA Genes in Alzheimer's Disease
Source: PLoS One. 2011 Jul 22;6(7):e22585. doi: 10.1371/journal.pone.0022585 (PMC3142181; doi:10.1371/journal.pone.0022585)
Supplement: Table S1 — Primers and PCR conditions. (DOC) [file pone.0022585.s006.doc]

**Table S1: primer sequences and PCR conditions.**

| **Reaction** | **Primer sequence**  **(5’ → 3’)** | **Amplicon length (bp)** | **Annealing temp. for PCR (°C)** | **MgCl_2_ conc. (mM)** | **Primer conc. (nM)** |
| --- | --- | --- | --- | --- | --- |
| Bisulfite fwd | GTGTGTGTTTTTGGGTTGATTAGAG | 247 | 57 | 1.5 | 100 |
| Bisulfite rev | CATCCAAAAACCCAACCTCTCC |
|  |  |  |  |  |  |
| Genomic fwd | GAGTGTGTCCCGGTCGTAGGAG | 487 | 63 | 1.5 | 100 |
| Genomic rev | CACCGGGAGTCGGGACGCTC |
|  |  |  |  |  |  |
| 18S rRNA fwd | TCAACTTTCGATGGTAGTCGCCGT | 108 | 60 | NA | 100 |
| 18S rRNA rev | TCCTTGGATGTGGTAGCCGTTTCT |
|  |  |  |  |  |  |
| 5.8S rRNA fwd | GACTCTTAGCGGTGGATCAC | 79 | 60 | NA | 100 |
| 5.8S rRNA rev | TCCTGCAATTCACATTAATTCTCG |  |  |  |  |
|  |  |  |  |  |  |
| 28S rRNA fwd | TTAAGCATATTAGTCAGCGGAGG | 75 | 60 | NA | 100 |
| 28S rRNA rev | GCTCTTCCCTGTTCACTCG |  |  |  |  |
|  |  |  |  |  |  |
| 18S genomic fwd | AGCCTGAGAAACGGCTACCA | 64 | 60 | NA | 100 |
| 18S genomic rev | GGTCGGGAGTGGGTAATTTGC |
|  |  |  |  |  |  |
| K-CTT fwd | CTAGCTCAGTCGGTAGAGCATG | 63 | 60 | NA | 100 |
| K-CTT rev | CCAACGTGGGGCTCGAAC |
|  |  |
| Met-HpaII fwd | GTATATCTTTCGCTCCGAGTCG | 73 | 60 | NA | 100 |
| Met-HpaII rev | ACAGGTCGCCAGAGGACAG |  |  |  |  |
