# Supplementary material for: Epigenetic Silencing of Nucleolar rRNA Genes in Alzheimer's Disease
Source: PLoS One. 2011 Jul 22;6(7):e22585. doi: 10.1371/journal.pone.0022585 (PMC3142181; doi:10.1371/journal.pone.0022585)
Supplement: Methods S1 — A text file containing detailed description of the modified SAM statistics. (DOC) [file pone.0022585.s007.doc]

**Methods S1**

*SAM Analysis.* Under the site methylation model it is hypothesized that the particular set of sites within the CpG island is the one responsible for gene silencing. Under this hypothesis the statistical analysis across AD and control groups may be conducted in a manner similar to the gene expression analysis on microarray where the ultimate goal is to identify genes which are most likely the “drivers” of different expression profiles across conditions. We applied a similar idea to the identification of methylation “driver” sites across two groups. Specifically, in order to identify the most likely differentially methylated sites modified version of the SAM model (Tusher et al., 2001) developed for permutation-based expression analysis was applied. The modification that we applied is based on the premise that CpG islands on the same promoter are likely correlated, with ones in closer locations having stronger associations. The adjustment of the SAM permutation model is therefore based on the application of a local polynomial regression (Cleveland, 1981) model across CpG sites, which allows one to compare local averages of methylation levels pooled across neighboring locations. In all models the smoothing parameters for the local regressions were selected via 5-fold cross-validation as described e.g., in Eubank (1999). The 95% confidence bounds for the regression trend lines are obtained by applying local regression to the permutation distribution of the methylation levels; the permutation distribution is obtained by enumerating all possible selections of 10 subjects into AD group (or equivalently, the remaining ones into control group). For the current groups sizes (n=10 or 5 in each group for the prefrontal cortex or cerebella, respectively) a total of over 180,000 with n=10, or 252 with n=5 such distinct selections (subjects' arrangements into two groups; or "2n choose n") is possible; For n=10 the 5000 randomly drawn selections was out of the total number possible, whereas for n=5 the totality of all possible distinct arrangements was used to obtain the permutation distribution and the confidence bounds for the prefrontal cortex- or the cerebellar samples, respectively. The standard statistical software R (R Development Core Team 2009) was used to build and test the SAM models.

**References:**

Cleveland, W.S. (1981) LOWESS: A program for smoothing scatterplots by robust locally weighted regression. The American Statistician, 35, 54.

Eubank, R. L. (1999). Nonparametric Regression and Spline Smoothing, 2 Ed. New York: Marcel, Dekker, Inc. Pp.

Kalita, K., Makonchuk, D., Gomes, C., Zheng, J. J. and Hetman, M. (2008) Inhibition of nucleolar transcription as a trigger for neuronal apoptosis. *J Neurochem,* **105,** 2286–2299.

Tusher, V., Tibshirani, R., Chu, G. (2001) Significance analysis of microarrays applied to transcriptional responses to ionizing radiation. Proc. Natl. Acad. Sci. USA., 98:5116-5121, 2001.

R Development Core Team. (2009) R: A Language and Environment for Statistical Computing. R Foundation for Statistical Computing, Vienna, Austria.
